# A *Streptococcus suis* Harmonized Ide*_Ssuis_* Mac-1 Domain Genotype-Based Classification Proposal and Prevalence in European Isolates

**DOI:** 10.3390/vetsci13080807

**Published:** 2026-08-15

**Authors:** Vicky Fachinger, Isabel Steiner, Scott A. Callison, Hubert Gantelet, Kathrin Lillie-Jaschniski, Thomas Lewiner, Giovani Trevisan

**Affiliations:** 1 Ceva Innovation Center, 06861 Dessau, Germany; 2 Ceva Bestvac, 06861 Dessau, Germany; 3 Ceva Animal Health, LLC, Shawnee, KS 66215, USA; 4 Ceva Biovac, 49070 Beaucouzé, France; 5 Ceva Santé Animale, 33500 Libourne, France; 6 Veterinary Diagnostic and Production Animal Medicine, Iowa State University, Ames, IA 50011, USA

**Keywords:** *Streptococcus suis*, Ide*_Ssuis_*, Mac-1, genotyping, immunity, classification, immunoglobulin M-degrading enzyme

## Abstract

The classification systems currently in use for *Streptococcus suis* are primarily based on capsular polysaccharide (CPS) serotyping or multi-locus sequence typing (MLST). A genetic typing system based on the conserved Mac-1 domain of the Ide*_Ssuis_* gene, which encodes an IgM-cleaving protease, could complement these approaches. Specifically, in this study, a genotyping system based on Mac-1 sequence homology was established, qualitative SYBR Green real-time PCR (qPCR) assays for standardized genotype classification were developed, and the global and European distribution of genotypes were analyzed. The Mac-1 classification system identified “two genotypes”, one of which has “two subgenotypes”, based on 427 unique sequences obtained from 4161 global sequences. GT1.1 was the most prevalent, having an amino acid similarity of 97.1%, followed by GT1.2 98.1%, and GT2 91.4%. The qPCR approach enabled reproducible, cross-laboratory-accessible typing. Among 5479 *Streptococcus suis* European isolates, 64% were GT1.1, 4.5% were GT1.2, and 29.6% were GT2, with a trend toward increasing GT1.1 detection rates between 2018 and 2023. Genotyping classification could complement current *Streptococcus suis* classification approaches to better support vaccine design and disease control strategies.

## Simple Summary

*Streptococcus suis* is a bacterial pathogen in pigs that poses challenges for accurately classifying clinically relevant strains with current methods, hindering the prediction of disease risk and the development of effective vaccines. This study aimed to improve classification by examining the conserved Mac-1 domain of a specific enzyme (Ide*_Ssuis_*) used by *Streptococcus suis* to specifically degrade porcine IgM, propose a genotyping classification, develop a qualitative SYBR Green real-time polymerase chain reaction (qPCR)-based test to group strains into genotypes, and analyze the prevalence of these groups in Europe. Two main genotypes (GT1 and GT2) were identified, with one of them having two subgenotypes, i.e., GT1.1 and GT1.2. GT1.1 was the most common, accounting for about 64% of European strains. The new qPCR method avoids the need for expensive sequencing technology and can be easily used across laboratories to differentiate GT1 from GT2 and GT1.1 from GT1.2, providing a more practical way to monitor bacterial variation. Ultimately, this approach could complement current *Streptococcus suis* classification methods to better support vaccine design and disease control strategies, helping protect pig health and reduce economic losses in the livestock industry.

## 1. Introduction

*Streptococcus suis* (*S. suis*) is a Gram-positive bacterial pathogen that commonly colonizes the tonsils and upper respiratory tract of pigs [1] without causing disease [2]. *S. suis* also has saliva as a common habitat [3]. The bacterium has a worldwide distribution, and factors such as stress or co-infections can enable virulent strains to trigger clinical illness. Diseases associated with *S. suis* are an increasing concern for swine health and production [4,5] and typically include meningitis, septicemia, arthritis, endocarditis, and sudden death [6]. *S. suis* is the leading bacterium in confirmed porcine disease diagnosis cases in the United States [7] and is commonly detected in nursery-age pigs and is the predominant pathogen co-diagnosed in respiratory cases during the nursery and finishing phases [8]. The cost of *S. suis*-associated diseases, including morbidity, mortality, antimicrobial treatment, and vaccination costs per pig in an affected farm has been estimated at between €0.6 and €1.3 in Europe and between €0.67 and €1.51 in the United States, with a mean of €1.08 per pig ($1.25; 95% CI: $0.77–$1.74) [9,10]. *S. suis* is also recognized as a zoonotic pathogen capable of causing serious disease in humans, typically through occupational exposure [11]. The need for *S. suis* antimicrobial treatments in livestock farming, as well as the potential for antimicrobial resistance, are also public health concerns [10].

Serotyping is the most widely used method for differentiating *S. suis* strains based on the antigenicity of capsular polysaccharides (CPS). The antigenicity of CPS is considered to be a major virulence factor and was originally used to classify *S. suis* into 35 serotypes (1–34 and 1/2) [12,13,14,15]. However, six of the serotypes (20, 22, 26, 32, 33, and 34) have been reclassified as belonging to other bacterial species [16,17,18]. Accordingly, 29 *S. suis* serotypes (1–19, 21, 23–25, 27–31, and 1/2) are currently recognized [4] and may be relevant to swine. Nevertheless, the majority of *S. suis* strains isolated from diseased pigs belong to serotypes 1–9 and 1/2 [11]. Due to the clinical relevance of serotyping, autogenous vaccine development exploits serotype-specific protection conferred by distinct CPS, which appears to be homologous protection [19]. Importantly, autogenous vaccine candidate strains should be isolated from systemic sites, such as the meninges, spleen, liver, and joints, rather than from potential commensal sites, such as the lungs, nasal cavities, or tonsils [6].

A less commonly used typing system for *S. suis* strains is based on sequence type(s) (ST), developed to characterize clonal relationships between strains and the potential of particular clones to cause disease [20]. An ST is determined by multilocus sequence typing (MLST), which is based on nucleotide sequences of seven *S. suis* housekeeping genes (*aroA*, *cpn60*, *dpr*, *gki*, *mutS*, *recA*, and *thrA*). For each gene fragment, the different nucleotide sequences are assigned to allele numbers, and the ST of each strain is defined by the alleles present at each of the seven distinct loci. Strains that share the same ST are assumed to be members of the same clone or to have a recent common ancestor, and clonal complexes may be defined based on shared alleles across multiple loci. Members of a clonal complex were defined as a group of two or more independent strains that shared identical alleles at 5 or more loci [20]. However, classification based on ST alone may be limited by the high level of genetic diversity of *S. suis*, with up to 3562 *S. suis* STs having already been identified in PubMLST (Public Databases for Molecular Typing and Microbial Genome Diversity, https://pubmlst.org/ accessed on 22 July 2026). Whether any of these housekeeping genes have protective properties as vaccine antigens has not been investigated to date [21]. It should, however, be noted that the housekeeping genes used to determine STs are not expressed on the *S. suis* bacterial surface and are most likely not involved in the natural immune response against *S. suis* infection. Furthermore, there is no correlation between serotype and ST, i.e., a serotype can be identified across different STs, but also certain serotypes can be associated with specific STs [21].

**Figure 1:**
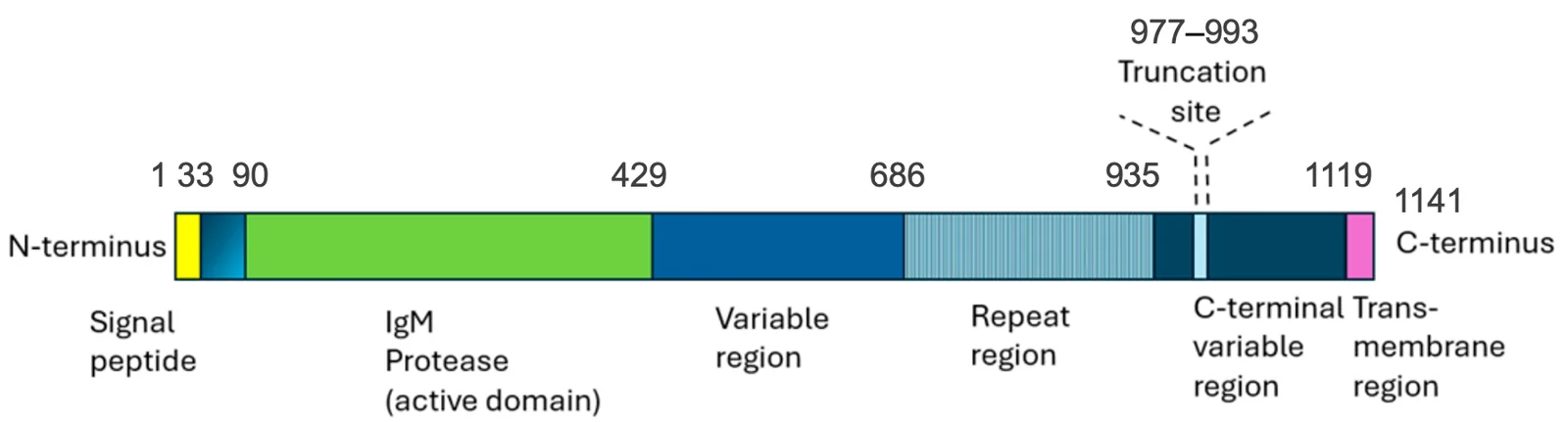
Graphical schematic representation of the structure of the Ide*_Ssuis_* protein (*S. suis* strain 10, GenBank SRR11931227).

The Immunoglobulin M (IgM) degrading enzyme, hereafter named Ide*_Ssuis_*, shows close homology to the IgG-specific endoprotease IdeS of *S. pyogenes* (also known as Mac-1) [22,23]. Due to this genetic homology, Ide*_Ssuis_* belongs to the IdeS endoprotease family [23]. The function of Ide*_Ssuis_* in *S. suis* infections differs, however, fundamentally from that of other endoproteases of the IdeS family, which degrade IgG solely. In contrast to this, Ide*_Ssuis_* cleaves intact IgM multimers from pigs’ IgM at domains 2 and 3 [22] but not from other species, such as humans, cattle, mice, cats, dogs, or closely related members of the *Suidae* family (warthog) [23]. Also, Ide*_Ssuis_* cannot degrade porcine or human IgG or IgA. Accordingly, Ide*_Ssuis_* of *S. suis* is, to date, the only known protease that cleaves intact IgM multimers [22] and indicates the functional adaptation of *S. suis* to pigs as a host [23]. By using the *S. suis* strain 10 (GenBank SRR11931227) as a reference strain [24], the Ide*_Ssuis_* protein can be characterized as having 1141 amino acids (aa) in length and consists of a signal peptide, the Mac-1 active domain, a variable region including a repeat region (with no similarity to Ide*S*) and a transmembrane region (see Figure 1). The Mac-1 active domain of the IgM protease is highly conserved and is composed of the 339 aa positioned between aa 91 and 429 with similarity to the IdeS active domain near the end of the aa motif EYFN.

The ever-evolving genetic diversity of *S. suis* and the need to better understand the epidemiological occurrence and clinical relevance of different isolates create an opportunity to explore additional *S. suis* genes of relevance and to develop genetic-based classification approaches. The serotyping system is based on the CPS, whereas the ST is determined through sequencing of seven housekeeping genes. Neither CPS serotype nor ST directly captures surface-exposed antigenic targets. In contrast, the Ide*_Ssuis_* antigen is surface-associated or secreted and is not part of the CPS-based serotyping system or the ST scheme. Accordingly, these available classification systems do not fully capture antigenic features that may be relevant to vaccine-induced protection. However, vaccine strategies will likely rely on conserved *S. suis* antigens being able to provide broad cross-protection across different serotypes. As a suitable candidate, recombinant Ide*_Ssuis_* has been identified [22], which may be efficacious for some genetic groups but not for others. It was therefore the objective of this study to characterize *S. suis* strains based on Ide*_Ssuis_* Mac-1 active domain sequence homology, propose a genotyping classification approach, develop a qPCR-based genotyping test, and assess the prevalence of Ide*_Ssuis_*-based genotypes in European countries.

## 2. Materials and Methods

### 2.1. S. suis Genetic Strains and Mac-1 Domain Sequence Collection and Curation

To provide an overall, worldwide snapshot of the diversity of *S. suis* based on the Mac-1 domain, i.e., the active domain of the Ide*_Ssuis_* gene over time, a four-step approach was used: (a) *S. suis* sequences were searched and retrieved from the NCBI GenBank protein database; (b) complete *S. suis* sequences were searched and retrieved from the NCBI GenBank database; (c) a total of 91 *S. suis* sequences recently published by a previous study [25] were added; and (d) a total of 43 *S. suis* sequences available from a private database and provided by two autogenous vaccine companies, Biovac (CEVA Biovac, Angers, France) and Bestvac (CEVA Bestvac, Dessau, Germany), were included in the analysis. The *S. suis* sequences recovered from GenBank and used in this study had collection years, when provided, ranging from 1981 to 2023.

Since the Mac-1 domain is part of Ide*_Ssuis_* the initial genetic sequence collection procedure was designed to recover the Ide*_Ssuis_* sequences followed by the identification of Mac-1. GenBank publicly available Ide*_Ssuis_* sequences can be retrieved in different forms, either properly annotated in the protein database or as part of a larger *S. suis* sequence covering the full or partial genome of the isolate. Ide*_Ssuis_*-annotated protein sequences were retrieved from NCBI GenBank in 2024. When available, metadata for sequences were retrieved to provide information on sequences derived from porcine or other species (e.g., humans) with and without clinical disease, as well as from different serotypes, STs, and geographic regions.

At the time of collection, the raw sequence reads were assembled using the Read Assembly and Annotation Pipeline Tool (RAPT), which is no longer available, from NCBI for assembly, nucleotide-to-protein translation, and annotation of the Ide*_Ssuis_* protein sequences, using the Ide*S* [26] gene as a reference. Full-length *S. suis* sequences with incomplete annotations were identified using conserved motifs. Their identification was then validated by homology comparisons with previously identified endoprotease IdeS of *S. pyogenes* (also known as the Mac-1 virulence factor), which is present in human- and pig-derived isolates, and was used as a reference in Geneious Prime (Geneious Prime 2026.0.1, https://www.geneious.com).

**Figure 2:**
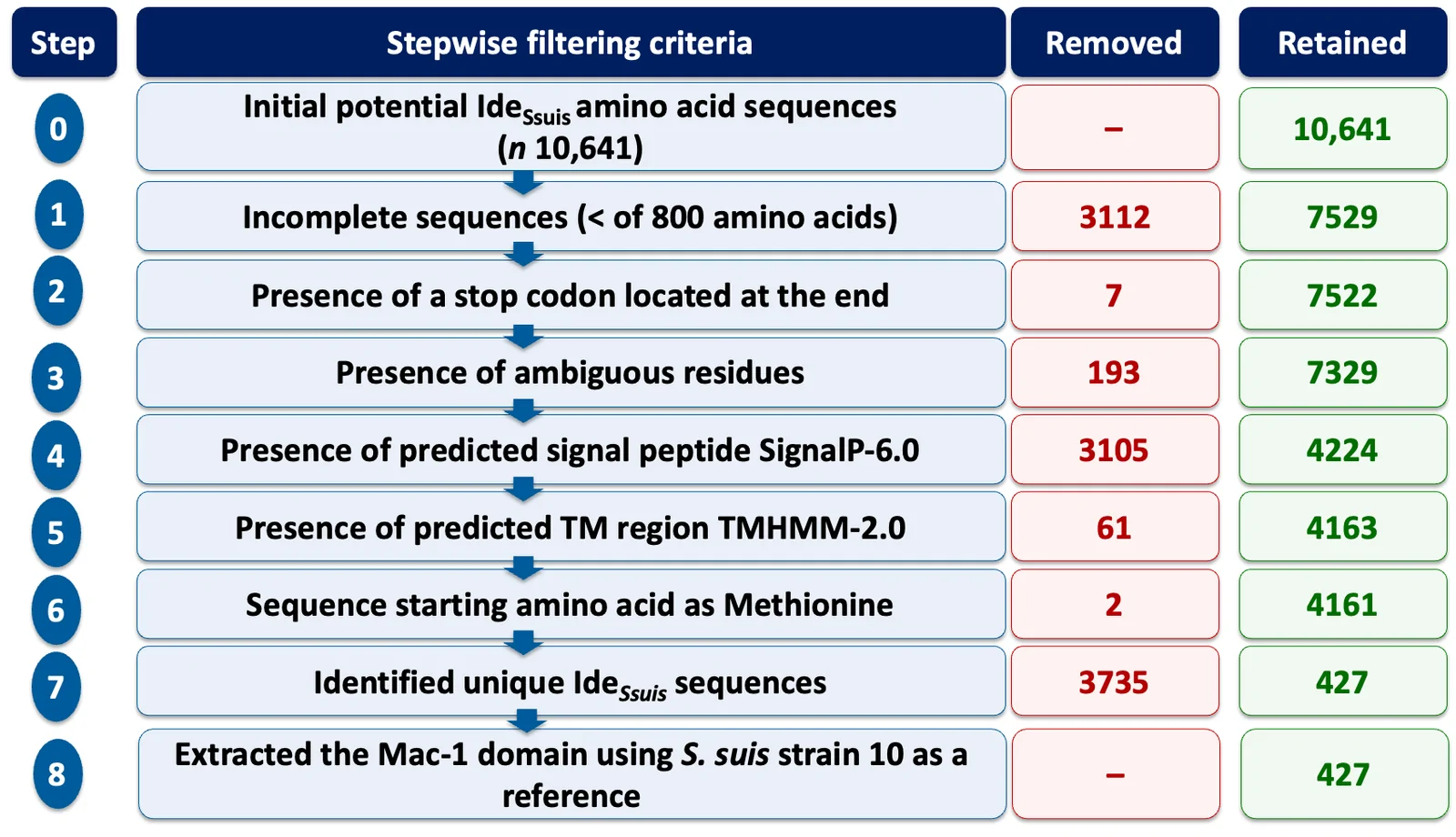
Stepwise flowchart for Ide*_Ssuis_* data curation and identification of Mac-1 domain sequences.

*S. suis* sequences were further curated to identify unique Ide*_Ssui_*_s_ sequences for subsequent Mac-1 domain extraction and genotyping classification evaluation. *S. suis* sequences collected from GenBank (*n* = 10,507), the sequences reported in another study [25] (*n* = 91), and private sequences (*n =* 43) were further processed and combined for Ide*_Ssuis_* gene identification. From an initial set of 10,641 Ide*_Ssuis_* amino acid sequence candidates, a stepwise filtering process was applied to identify high-confidence Ide*_Ssuis_* sequences (Figure 2). Sequences were sequentially excluded if they: (a) were incomplete, defined as shorter than 800 aa; (b) lacked a stop codon at the end of the coding sequence; (c) contained ambiguous residues; (d) lacked a predicted signal peptide, as determined by SignalP-6.0 [27] (SignalP-6.0, https://services.healthtech.dtu.dk/services/SignalP-6.0/ accessed 10 May 2026); (e) lacked a predicted transmembrane (TM) segment, as determined by TMHMM-2.0 [28,29]; (f) lacked an N-terminal methionine; or (g) duplicated sequences which were then removed to retain unique entries. The Mac-1 domain was subsequently identified and extracted from each sequence using the *S. suis* strain 10 as a reference [24], resulting in a final set of 427 Mac-1 domain sequences.

To identify unique Ide*_Ssuis_*, the aa sequences were pairwise aligned using MAFFT [30], and duplicates were removed, keeping only unique Ide*_Ssuis_*. To identify the active Mac-1 domain present in the unique Ide*_Ssuis_* sequences, the *S. suis* strain 10 (GenBank SRR11931227 [24]) Mac-1 domain, composed of 339 aa positioned between aa 91 and 429 of Ide*_Ssuis_*, was used as a reference. The Mac-1 domain of *S. suis* strain 10 was annotated to the 427 uniquely identified Ide*_Ssuis_* sequences; the annotated portion was extracted and pairwise aligned using the MAFFT algorithm [30], and the phylogeny was constructed and displayed using an RAxML phylogenetic tree [31]. All the steps mentioned here were performed using default settings in Geneious Prime (Geneious Prime 2026.0.1, https://www.geneious.com). Sequence visualization was displayed in a phylogenetic tree built using RAxML standard procedures available in Geneious Prime.

An additional step was taken to identify Ide*_Ssuis_* variable regions. The Ide*_Ssuis_ S. suis* 10 strain regions were annotated as N-terminus and a signal peptide composed of 90 aa positioned between aa 1 and 90, the Mac-1 domain (previously described), variable region 256 aa positioned between aa 430 and 686, repeated region 249 aa positioned between aa 686 and 935, C-terminal variable region 185 aa positioned between aa 934 and 1119, and transmembrane region 21 aa positioned between aa 1120 and 1141. The unique 427 strains were pairwise aligned using the MAFFT algorithm [30], and each genome region was extracted based on the alignment in Geneious Prime. Pairwise aa similarity was calculated for each region: N-terminus and signal peptide, Mac-1 domain, variable region, repeated region, C-terminal variable region, transmembrane region, and the full-length Ide*_Ssuis_*, producing a symmetric pairwise similarity matrix for each region. Summary statistics (minimum, first quartile, median, mean, third quartile, maximum, and number of pairwise comparisons) were calculated for each region, and analyses were performed in R (version 4.4.2; R Core Team, 2025) using the tidyverse package suite [32].

### 2.2. Characterization and Determination of Mac-1 Domain Genotypes

The Mac-1 domain, the active domain of Ide*_Ssuis_*, served as a basis for genotype and subgenotype classification. Multiple sequence alignments of the 427 Mac-1 domain aa sequences were performed using MAFFT [30] using default parameters, and a neighbor-joining phylogenetic tree was constructed using an RAxML format [31] with bootstrap support from 50 replicates. Phylogeny display was used to identify distinct groups. Clusters of sequences with pairwise distances between groups exceeding 10% were assigned to different groups and designed as genotypes. The genotype with the largest number of sequences was further divided into subgenotypes, each defined as having a mean aa similarity of at least 97% within the group. Tree visualization was performed in Geneious Prime (Geneious Prime 2026.0.1, https://www.geneious.com). Genotype and subgenotype assignment criteria were selected based on the distribution of sequence distances observed in the dataset and the phylogenetic separation of the identified clusters.

### 2.3. Characterization and Prevalence Determination of Mac-1-Based Genotype of Strains Recovered from Pigs in Europe Between 2018 and 2023

#### 2.3.1. *S. suis* Isolates Source

To identify the Mac-1 genotype and subgenotype and estimate the detection prevalence recovered in Europe, a private collection of *S. suis* isolates was used. A total of 5479 isolates were collected between 2018 and 2023 and recovered from field samples intended for autogenous vaccine production. *S. suis* isolates were provided by Biovac (*n* = 4368) (CEVA Biovac, Angers, France) and by Bestvac (*n* = 841) (CEVA Bestvac, Dessau, Germany). The isolates were recovered from Europe, and Table 1 summarizes the geographic and temporal distribution of these European isolates, including the number of isolates per country and year of collection.

After recovery from field samples, the isolates were kept in long-term storage until the start of the study. All isolates were stored at −80 °C, either as glycerol stocks or using the CRYOBANK Cryopreservation System (Mast Group, Liverpool, United Kingdom). The isolates were thawed and streaked onto Columbia Agar with Sheep Blood, order no. PB5008A (Thermo Fisher Scientific, Waltham, MA, USA), for cultivation and incubated overnight at 37 °C in an incubator. The *S. suis* isolates were recovered from sample types (e.g., joints, brain, spleen, lungs, etc.) of pigs deemed clinically sick, but no final confirmation of *S. suis* as the causative agent for a disease diagnosis was available. Ide*_Ssuis_* detection was reported over time by year, country, serotype, and sample type compared with the proposed genotypes.

#### 2.3.2. Procedure for Qualitative SYBR Green Real-Time PCR (qPCR) Development and Testing of European Isolates for Genotyping Differentiation

To characterize the genotype or subgenotype present in these isolates, real-time quantitative polymerase chain reaction (qPCR) assays targeting Ide*_Ssuis_* were developed and used to test all isolates and determine the prevalence of different genotypes in the culture isolates. Ide*_Ssuis_* gene sequencing was not performed in the isolates used for the European prevalence study. For DNA preparation, *S. suis* strains were picked from an agar plate and transferred to a 96-well PCR plate containing 50 µL/well of the Instagene Matrix (BioRad, Hercules, CA, United States). To separate DNA from bacterial proteins, the sealed plates were incubated at 56 °C for 20 min, then at 96 °C for 8 min. Thereafter, the plates were centrifuged for 3 min at maximum speed, and the DNA-containing supernatant was transferred to a new PCR plate, while the protein-containing matrix remained in the original plates. Procedures were performed according to the manufacturer’s instructions. The PCR plates containing DNA were sealed and stored at −20 °C until further use. For differentiation of *S. suis* genotype 1 (GT1) and genotype 2 (GT2), two qPCRs were developed using primers binding to the region coding for the IgM protease part, i.e., the Mac-1 domain, of the Ide*_Ssuis_* gene (see Table 2). Further, a third qPCR was developed to determine whether an Ide*_Ssuis_* of GT1 belongs to the subgenotype GT1.2 using primers binding the variable region downstream of the IgM protease motif (see Table 2). The fragment size to which the respective primer pairs were bound was 130 bp for GT1, 173 bp for GT1.2, and 107 bp for GT2. The strains used as a positive control for qPCR development included: (a) GT1.1: strain 16085/3b, isolated from a spleen of a clinically diseased pig in Germany in 2016 [33] (GenBank SRR11931223) [24]; (b) GT2: isolate GD-0034, recovered from cerebrospinal fluid or blood of a pig in the Netherlands (GenBank NZ_CZFM01000001.1); and (c) GT1.2: strain 4-1-1-019, isolated from a clinically diseased pig with no site of recovery identified in Germany in 2016 by Dr. Schliephake, LA Sachsen Anhalt, Germany (in-house sequenced). DNA amplification was carried out in a 20 µL final reaction volume containing 10 µL of the kit-supplied SYBR Green PCR master mix (Sso Advanced Universal SYBR Green Supermix, Biorad, Hercules, CA, United States); 2 µL of the primer mix for either GT1, GT1.2, or GT2 (2.5 µM); 6 µL of nuclease-free water; and 2 µL of the DNA sample. The thermal profile for the qPCR was 98 °C for 3 min (for activation and DNA denaturation), followed by 30 cycles at 98 °C for 15 s (for denaturation) and 60 °C for 30 s (for annealing and amplification). Subsequent melting curve analysis was performed by heating the PCR reactions from 65 °C to 95 °C (0.5 °C increments every 5 s). Fluorescence of the samples was continuously monitored as the temperature increased. SYBR Green I fluoresces upon binding to double-stranded DNA, and fluorescence decreases as DNA is denatured. For analytical validation of the qPCR assays, each run included three wild-type *S. suis* control strains representative of GT1.1, GT1.2, and GT2, together with a nuclease-free water non-template control. The non-template controls remained negative. Samples were considered positive if they showed a sigmoidal amplification curve and a Ct value less than 30, and genotype assignment was performed according to the decision matrix presented in Table 3. Isolates without amplification in any of the three assays were classified as non-typable; this group may comprise *S. suis* strains carrying Ide*_Ssuis_* variants outside the targeted regions as well as non-*S. suis* organisms, and future studies should therefore compare the Ide*_Ssuis_* qPCR genotyping with independent typing methods to further characterize these cases and establish analytical performance.

## 3. Results

### 3.1. Ide_Ssuis_ Sequence Dataset and Curation

For Mac-1 genotype identification an initial set of 10,641 Ide*_Ssuis_* protein sequences were obtained: 10,507 from GenBank, 91 independently published [25], and 43 internally derived sequences. After curation, reassembly, and data cleaning, 4161 sequences meeting the criteria for Ide*_Ssuis_* presence were identified. From the 4161, a final 427 unique Ide*_Ssuis_* sequences (Table S1) were identified and remained in the database for Mac-1 domain extraction and genotype identification (Figure 2).

**Figure 3:**
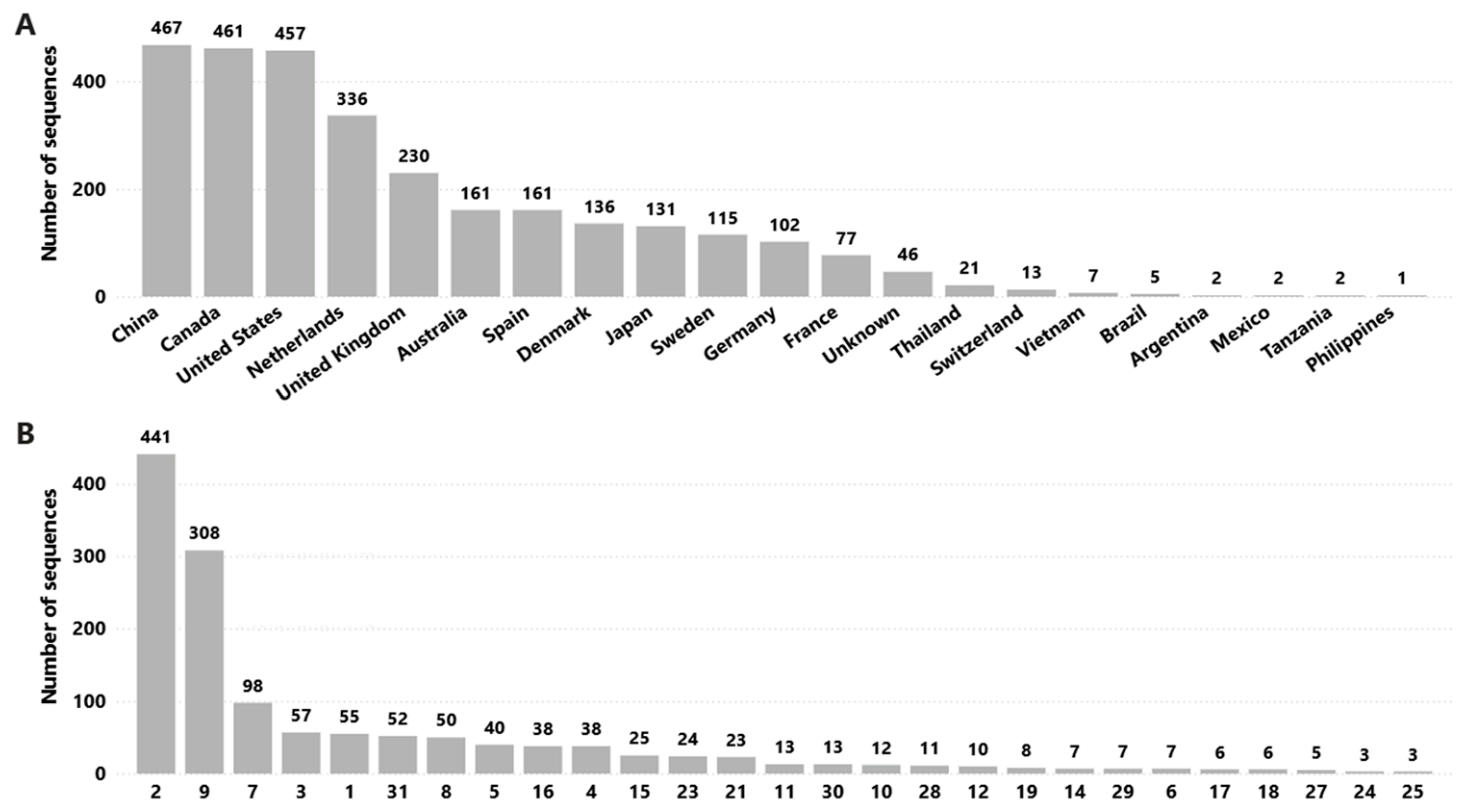
(**A**) Number of *S. suis* Ide*_Ssuis_* isolates recovered from pigs according to country of collection. (**B**) Number of Ide*_Ssuis_* recovered from pigs and the corresponding serotype.

The 4161 *Ide_Ssuis_* sequences were mostly recovered from pigs (70.5%, 2933 of 4161), with 24.9% (729 of 2933) identified as recovered from diseased pigs. The additional Ide*_Ssuis_* recovered sequences were from humans (8.4%, 348 of 4161), and one for each of the hosts: cat, dog, and fish. Another 21.1%, 877 of 4161, had no host identified. The 2933 Ide*_Ssuis_* recovered from pigs were identified as originating from 20 countries, with very similar numbers from China, Canada, and the United States (Figure 3A). Another 1.6% (46 of 2933) had no geographical location provided (Figure 3A). Of the 2933 Ide*_Ssuis_* recovered from pigs, 46.4% (1360 of 2933) had serotypes assigned, with serotypes 2 and 9 being predominant (Figure 3B). Across the final 427 unique Ide*_Ssuis_* strains, 63% (269 of 427) were recovered from pigs, 27.4% (117 of 427) had no host identified, 7% (30 of 427) were from humans, and 0.24% (1 of 427) was from fish.

### 3.2. Characterization and Prevalence Determination of Mac-1 Genotypes

**Figure 4:**
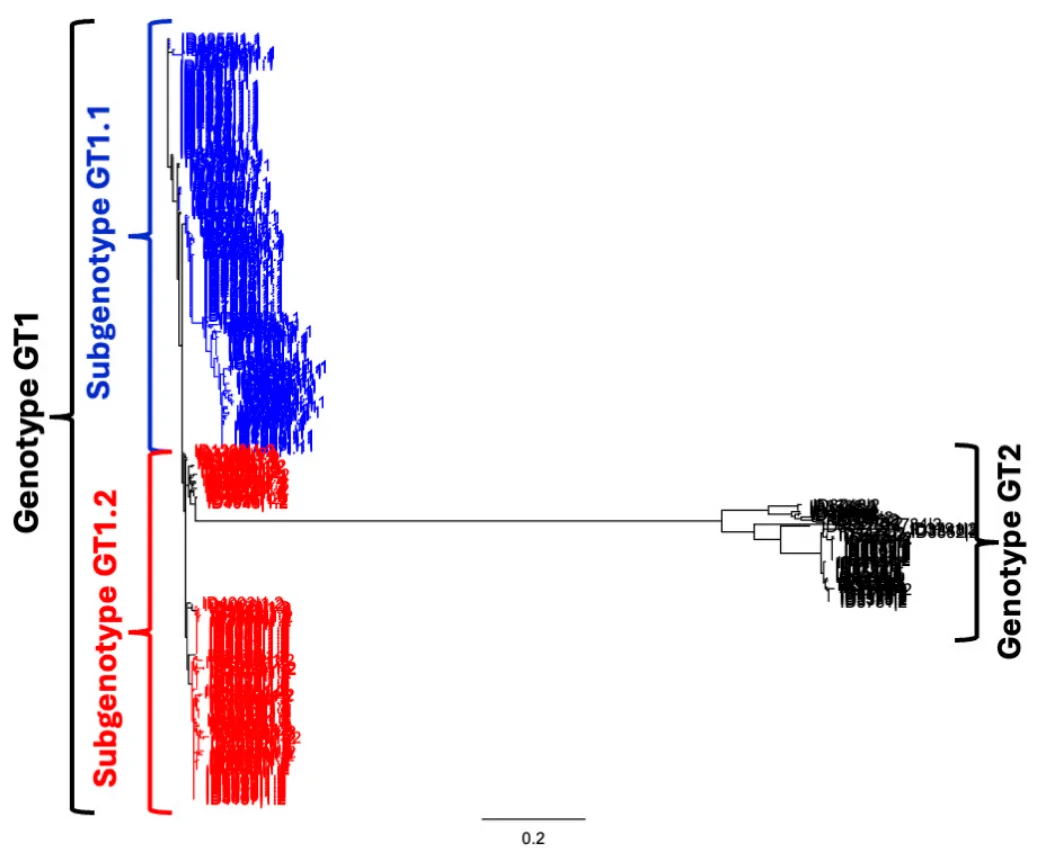
Neighbor-joining phylogenetic tree based on the Mac-1 domain sequences and corresponding genotypes. Mac-1 domains were extracted from the 427 unique full-length Ide*_Ssuis_* strains recovered from 1981 to 2023. Genotype groups are indicated in the phylogenetic tree: (**left**) genotype 1 (GT1); (**right**) genotype 2 (GT2). Subgenotypes GT1.1 and GT1.2 are indicated in the tree. Left labels in the tree are genotype or subgenotype color-coded: blue: subgenotype GT1.1; red: subgenotype GT1.2; black: genotype GT2. Because of the high number of sequences, tree leaf labels schematically represent GT1.1, GT1.2, and GT2 and do not allow identification of individual sequence names.

Phylogenetic analysis of the 427 unique Mac-1 domains extracted from the Ide*_Ssuis_* collected worldwide from different species and health statuses revealed two distinct Mac-1 genotypes, designated as genotype 1 (GT1) and genotype 2 (GT2) (Figure 4). The homology between GT1 and GT2 was 62.2%, supporting the criteria for assignment as distinct genotypes. At the genotype level, based on the Mac-1 domain, the aa sequence identity was 96.8% within GT1 and 91.4% within GT2. Due to variability within GT1 sequences, two subgenotypes were identified: GT1.1 and GT1.2. At the subgenotype level, the homology within GT1.1 was 97.1%, and within GT1.2, 98.1%. The homology between GT1.1 and GT1.2 was 96.1% (Table 4). Although GT1.1 and GT1.2 shared high sequence similarity (96.1%), they formed distinct phylogenetic clusters, supporting their designation as separate subgenotypes. Across all retained Mac-1 sequences, 53.9% (230 of 427) belonged to GT1.1, followed by GT1.2 (33.3%, 142 of 427) and GT2 (12.9%, 55 of 427). The GT2 formed distinct clusters in the phylogenetic tree presentation.

Due to the limited number of sequences (55 of 427) belonging to GT2, no additional subgenotypes were proposed for GT2. When building the phylogenetic tree, the branch for GT2 is placed between GT1.2 sequences, giving the impression of two GT1.2 groups (Figure 4). The small cluster of GT1.2 sequences showed a within-aa similarity of 98.7%, and between GT1.1 and GT1.2, 96.7% and 97.6%, respectively. Because the small cluster sustained a >97% aa similarity threshold with GT1.2, the cluster was not further divided into an additional subgenotype.

The mean aa similarity of Ide*_Ssuis_* was 61.2%. The Mac-1 domain was the most conserved region, with a mean aa similarity of 88.7%, and the repeated region, the most variable, with a mean aa similarity of 32.3% (Table 5).

### 3.3. Characterization and Prevalence Determination of GT1 and GT2 in S. suis Isolates Recovered from Pigs in Europe Between 2018 and 2023

A total of 5479 isolates collected between 2018 and 2023 from European farms suspected of *S. suis* infection were further tested by qPCR for Ide*_Ssuis_* detection and genotyping. Across 79 independent runs, their Ct values and melting temperatures showed narrow dispersion (Table 6), with mean Ct values of approximately 18–21 and a single, reproducible melting peak at around 80 °C for GT1.1 and GT2 and 84 °C for GT1.2. Under the assay conditions used, positive control strains and positive test isolates typically yielded Ct values around 10–20, while negative controls showed no specific amplification. Melting curve analysis was used to assess product specificity; positive reactions showed a single melting peak at the expected temperature, with a variation of approximately ±2 °C, while negative controls did not display a specific peak.

**Figure 5:**
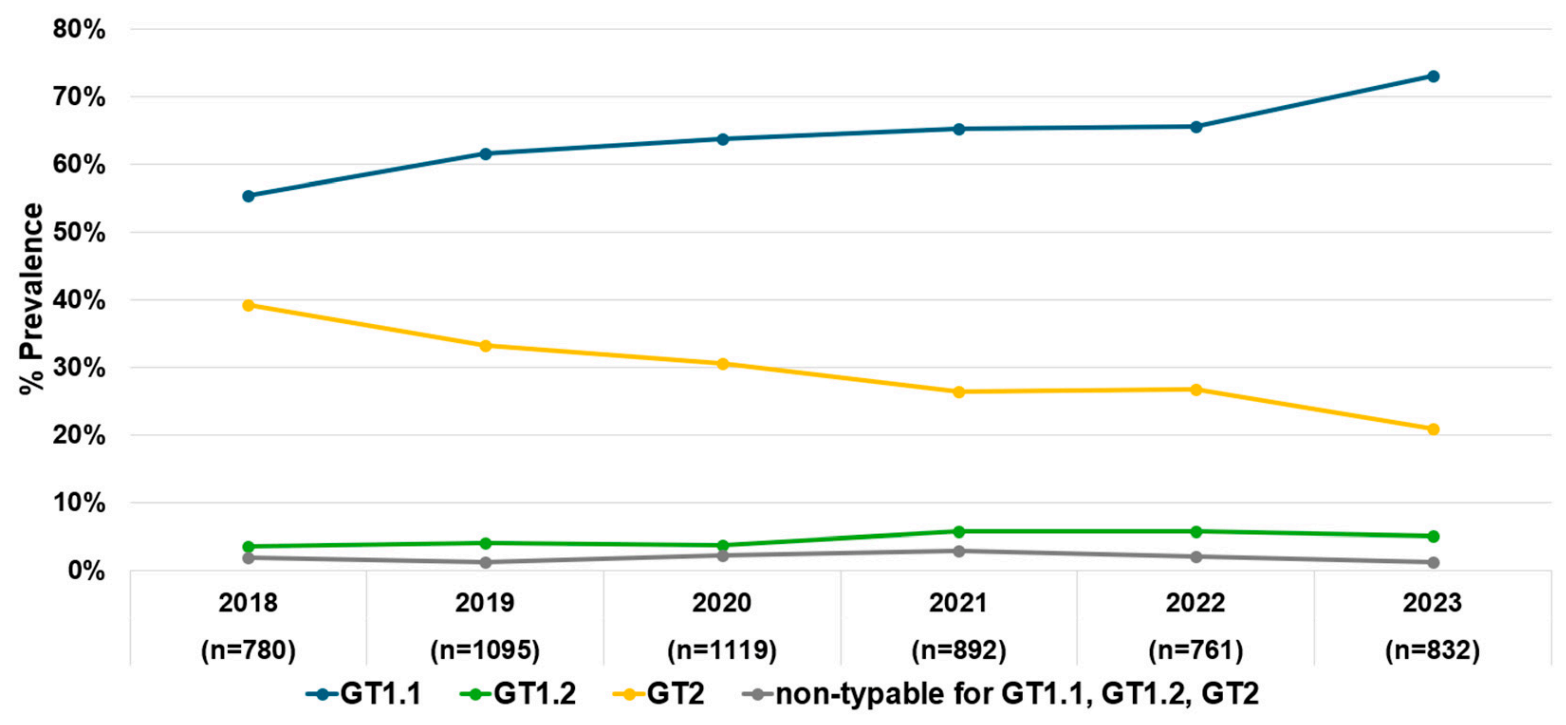
Prevalence of Mac-1 genotypes of European *S. suis* isolates over time detected by qPCR. The *x*-axis presents the year of collection, and the numbers below indicate the total number of isolates collected per year.

Based on the mean prevalence across isolates recovered from European countries, the GT2 prevalence was 29.6% (1622 of 5479), and GT1, 68.5% (3754 of 5479), or broken down to a subgenotype level, GT1.1 was 64% (3505 of 5479), and GT1.2, 4.5% (249 of 5479). A notable trend toward increased detection of GT1.1 was observed, rising from 55.5% (432 of 780) in 2018 to 73% (607 of 832) in 2023. On the other hand, a decrease in GT2 was observed, from 39.2% (306 of 780) in 2018 to 20.8% (173 of 832) in 2023 (Figure 5). GT1.2 remained similar across the years, representing 3.5% (27 of 780) in 2018 and 5% (42 of 832) in 2023. In a total of 1.9% (103 of 5479) of the strains, neither the GT1 nor the GT2 of the Ide*_Ssuis_* Mac-1 domain could be detected by qPCR (Ct values are above the threshold, with no or a non-specific melting curve).

The genotype distributions and temporal trends reported here should be interpreted with consideration of imbalances in geographic and temporal contributions to the dataset and sampling over time (see Section 2.3.1).

**Figure 6:**
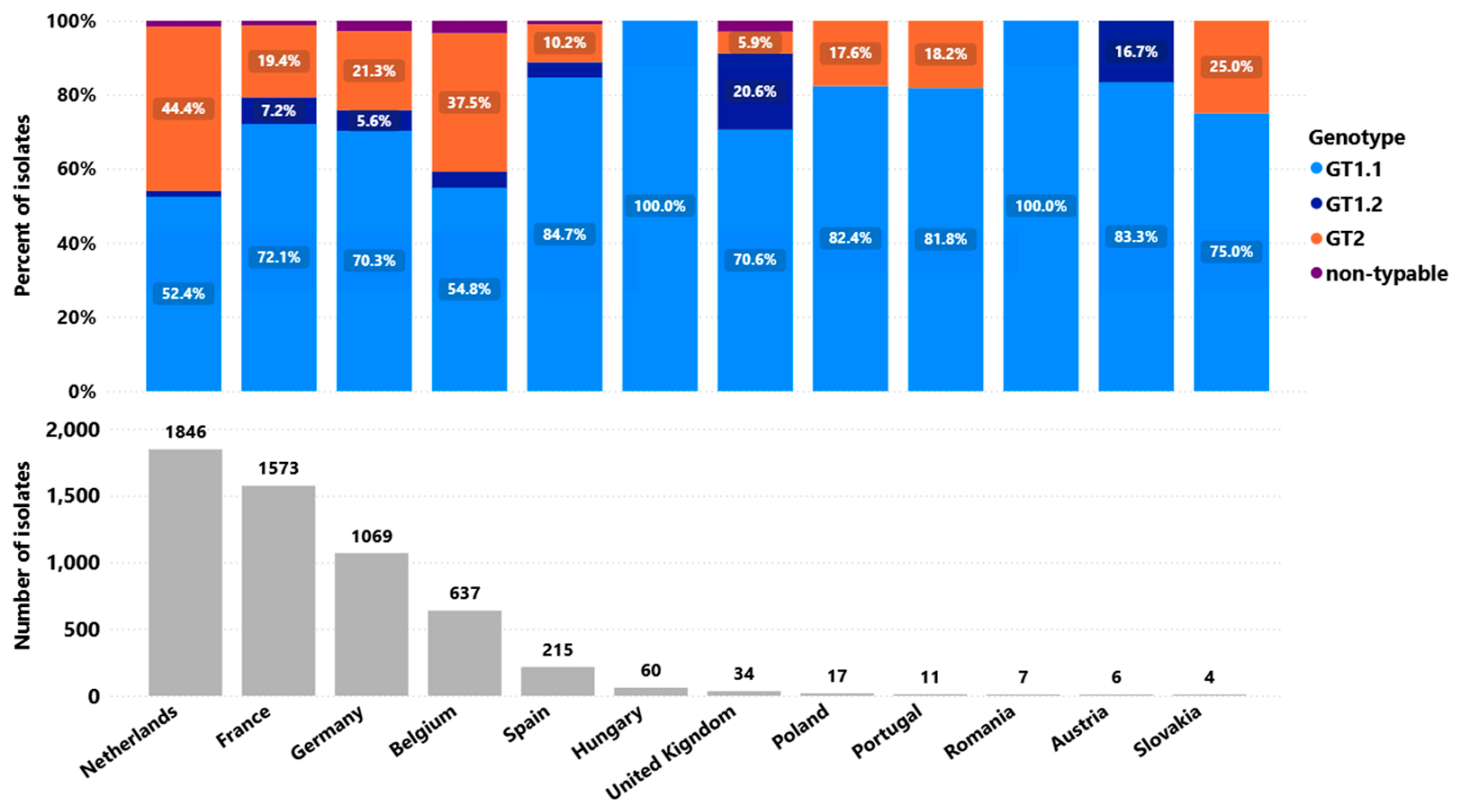
*S. suis* isolates tested by qPCR and broken down by genotype of the Mac-1 domain in European countries. The **bottom panel** shows the number of isolates by country. The **upper panel** shows the breakdown of percent genotypes by country. The numbers above the bars indicate the number of isolates and the percent of total isolates for each country.

The *S. suis* genotypes based on the Mac-1 classification and detected by qPCR were therefore also presented by country (Figure 6). The prevalence of GT1.1 and GT2 differed across countries. For countries with more than 20 isolates recovered, the highest prevalence of GT1.1 was found in Hungary (*n* = 60), where all isolates belonged to this subgenotype, followed by Spain (84.7%; 182 of 215), and the lowest were in the Netherlands (52.3%; 967 of 1846). In countries with fewer than 20 isolates recovered, all belonged to GT1.1 in Romania (*n* = 7), 82.4% (14 of 17) in Poland, 81.8% (9 of 11) in Portugal, and 83.3% (5 of 6) in Austria. The highest prevalence of GT2 was found in the Netherlands (44.4%; 820 of 1846) and Belgium (37.5%; 239 of 637), followed by Germany (21.3%; 228 of 1069), while in the remaining countries, the prevalence was below 20%, except for Slovakia, where 25% (1 of 4) isolates belonged to this genotype.

**Figure 7:**
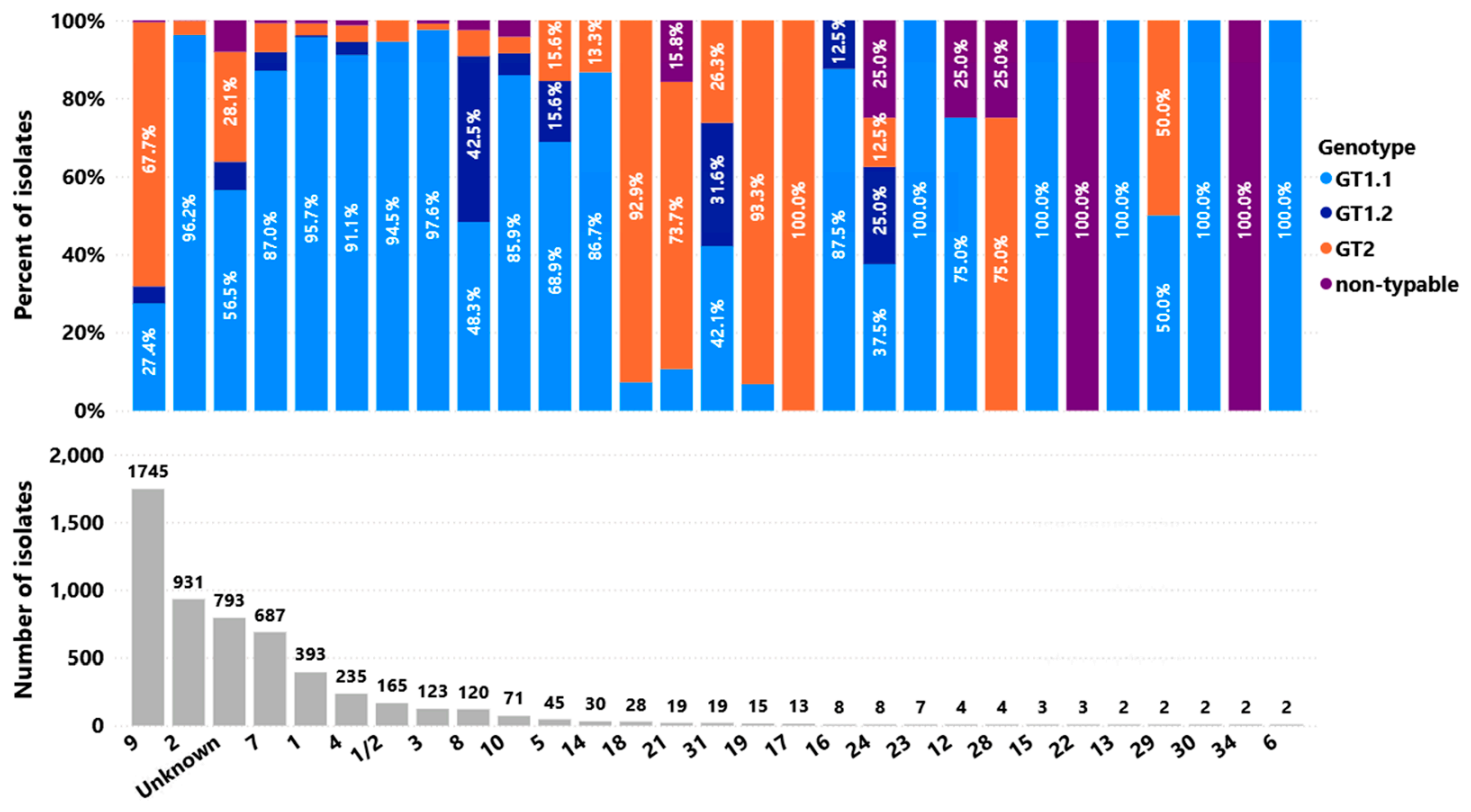
Presence of subgenotype 1 (GT1.1), subgenotype 2 (GT1.2), and genotype 2 (GT2) of the Mac-1 domain in different *S suis* serotypes. The **bottom panel** shows the number of *S. suis* isolates according to serotype. The **upper panel** shows the breakdown of genotypes per serotype. The numbers above the bars indicate the percent of total isolates for each serotype.

The Mac-1-based genotypes were not equally distributed among serotypes of *S. suis* isolates recovered from European countries. The GT1.1 detection was above 87% for serotypes 1–4 and 7 (Figure 7) and 68.9% for serotype 5. Additionally, GT1.1 detection accounted for all isolates of serotypes 6 (*n* = 2), 13 (*n* = 2), 15 (*n* = 3), 23 (*n* = 7), and 30 (*n* = 3) and accounted for 86.7% (26 of 30) of serotype 14 isolates. The highest prevalence of GT2 was found in serotype 17, where all 13 isolates belonged to this genotype, followed by serotype 19 (93.3%, 14 of 15), serotype 18 (92.9%, 26 of 28), serotype 28 (75%, 4 of 5), serotype 21 (73.7%, 14 of 19), and serotype 9 (67.7%, 1182 of 1745). In isolates with an unknown serotype, GT2 represented 28.1% (223 of 793). Across serotypes 1–8, GT2 accounted for <10%, except in serotype 5, where it accounted for 15.6% (7 of 45). This indicates that except for serotype 9, the major relevant serotypes in diseased piglets express GT1, with a prevalence > 90%. The proportion of the serotype 9 genotype GT2 differs markedly across countries and serotype datasets, with an overrepresentation in the Netherlands.

**Figure 8:**
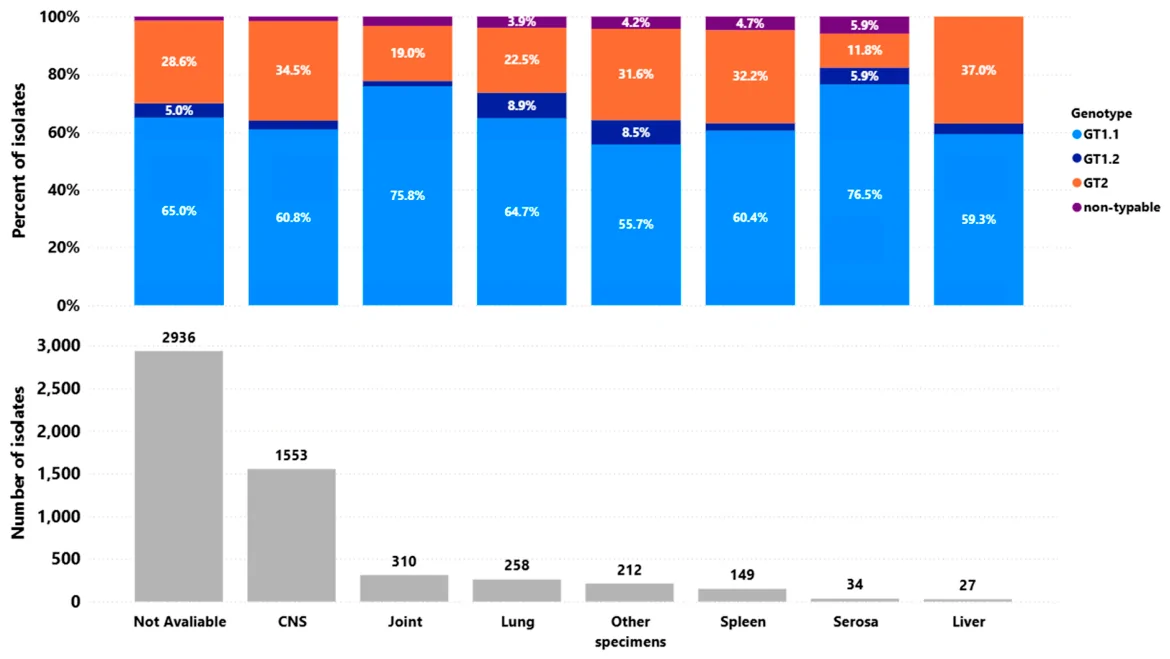
Presence of subgenotype 1 (GT1.1), subgenotype 2 (GT1.2), and genotype 2 (GT2) of the Mac-1 domain in different *S suis* isolates recovered from European countries and according to the site of isolation. The **bottom panel** shows the number of isolates by *S. suis* site of isolation. The **upper panel** shows the genotypes of the Mac-1 breakdown by site of isolation. The numbers above the bars indicate the percentage of isolates by genotype over the total for each site of isolation.

Information on the sampling organ was not available for 53.6% (2936 of 5479) of the samples (Figure 8). Specimens from the central nervous system (CNS), e.g., brain, cerebellum, and cerebrospinal fluid, accounted for 28.3% (1553 of 5479) of isolates. Detection of GT1 and GT2 was not equally distributed across sample types. For GT1.1, detection was higher in serosa (e.g., peritoneum, pericardium, and pleura) (76.5%, 26 of 34) and joints (75.8%, 235 of 310). In CNS specimens, GT1.1 accounted for 60.8% (945 of 1553). The highest detection of GT2 was in the liver (37%, 10 of 27), followed by the CNS (34.5%, 536 of 1553), and the lowest in the serosa (11.8%, 4 of 34).

## 4. Discussion

The analysis of 10,641 *S. suis* genetic sequences identified 4161 sequences meeting the criteria for Ide*_Ssuis_* presence and revealed 427 unique Ide*_Ssuis_* sequences, which were used to determine genetic differences and establish the basis for genotype classification based on the Mac-1 active domain. Two genotypes, i.e., genotype 1 (GT1) and genotype 2 (GT2), sharing a mean Mac-1 domain amino acid similarity of 62.2%, were identified. Genotype 1 (GT1) formed two distinct groups, phylogenetically separated from GT2 (Figure 4), and was further subdivided into subgenotypes 1 (GT1.1) and 2 (GT1.2), with 97.1% and 98.1% amino acid identity within each subgenotype and 96.1% between the two.

The Mac-1 domain, rather than the complete Ide*_Ssuis_* gene, was selected for the genotype classification system as it is the active domain, as well as the relevant and most conserved region of Ide*_Ssuis_* (Table 5). In contrast, the other regions of Ide*_Ssuis_* are more variable, which may have previously biased any analysis of the phylogenetic evolution of *S. suis* isolates.

The proposed genotype classification enables grouping and identifying genetically related sequences that contain a similar active IgM-degrading domain (aa 91–429; Mac-1 domain). Mac-1 is an enzymatically active IgM-cleaving region and functions as a catalytic domain that binds and cleaves porcine IgM [34,35]. IgM is the first antibody produced in the immunological response to *S. suis* infection [36], and IgM-cleaving activity can abrogate complement activation when IgM titers are high [37]. Therefore, Mac-1 domain-based selectin can help identify *S. suis* strains for selection and usage in vaccine development. In previous studies, it has in fact been shown that pigs vaccinated with recombinant Ide*_Ssuis_* and subsequently infected with *S. suis* isolates of serotypes 2 and 9 were protected from severe morbidity and exhibited reduced mortality [33,38]. As the underlying mechanism of protection, studies have shown that IgG antibodies are protective by inducing Fc-mediated opsonophagocytosis rather than neutralizing the IgM protease [27]. The proposed Mac-1 domain-based genotype classification enables further identification of Ide*_Ssuis_* strains within the same genotyping, aiding in characterizing *S. suis* strains and potentially enabling the selection of candidate strains for vaccine development.

The collection of sequences from GenBank can introduce biases, as certain research groups have been particularly active in *S. suis* research and have either provided relatively more sequences from their own country or sequences associated with a specific research question, which was, however, unrelated to Ide*_Ssuis_*. Despite this sampling bias, the dataset provides a broad representation of publicly available Ide*_Ssuis_* sequences from major pork-producing regions across most pork-producing countries, e.g., China, the United States, Canada, Japan, and European countries. In contrast, pork-producing countries like Brazil were underrepresented in this database.

The analyses of different Mac-1-based genotypes performed in this study used a large collection of 4161 Ide*_Ssuis_* available sequences representing 427 unique Ide*_Ssuis_*. A previous study developed Ide*_Ssuis_*-based genotyping using 2586 sequences representing 1999 unique Ide*_Ssuis_* [25]. Considerable methodological differences are presented in this study when compared with the previous study [25] (Table 7).

The findings encountered in this study differ from the previous study [25]. The genotype 1 (GT1), with subgenotypes GT1.1 and GT1.2, and genotype 2 (GT2) identified in this study are somewhat similar to the groups A, C, and B previously reported in the first study [25]. However, in this study, a proposal was made to classify strains by subgenotyping based on the degree of amino acid similarity of the Mac-1 domain (rather than the complete Ide*_Ssuis_* sequence), revealing considerable divergence between GT1 and GT2, with only 62.2% amino acid similarity. The aa similarity within the sequences of group GT1, corresponding to the previously reported groups A and C [25], was 96.8%, indicating a highly conserved group based on the Mac-1 domain and therefore supporting classification into a genotype with subgenotypes rather than different groups. The Mac-1 domain classification does not perfectly align GT1.1 with the previous group A [25], nor does GT1.2 align with group C [25], since some sequences flipped to the other Mac-1 genotype, e.g., some group A Ide*_Ssuis_* were classified as GT1.2 based on the Mac-1 domain and vice versa.

The distinction between GT1 and GT2 is strongly supported by amino acid identity values of 96.8% within GT1 and 91.4% within GT2, while amino acid identity between GT1 and GT2 is markedly lower at 62.2%. When using the Ide*_Ssuis_* classification from the previous study, groups A and C do not explicitly show such a relation [25]. Additionally, GT1.2, similar to group C [25], has been predominantly associated with isolates commonly recovered from healthy pigs and in serotypes less frequently associated with invasive disease, e.g., 31, 10, 27, and 24, rather than isolates associated with clinical disease, e.g., 9, 2, 7, and 1 [11,39,40,41,42,43]. Furthermore, GT1 was subdivided into two subgenotypes, GT1.1 and GT1.2, based on subtle but consistent differences in aa similarity. Although the overall similarity between GT1.1 and GT1.2 remains high, 96.1%, each subgroup exhibited higher internal identity with GT1.1 at 97.1% and GT1.2 at 98.1%, supporting their distinction as subgenotypes within GT1. GT1.1 was also the most prevalent among European isolates and among serotypes deemed pathogenic, such as serotype 2, making it a potential antigen candidate for consideration in selecting for vaccine development. The complementary use of Mac-1-based genotypes alongside serotyping and STs could result in better vaccine candidate selection; however, this evaluation was not performed under this study.

Among isolates recovered from European samples, GT1.1 showed a sustained increase over the last six years, suggesting a potential rise in prevalence. In contrast, GT1.2 remained at a consistently low level over time, whereas GT2 decreased. GT1.1 was also the most abundant genotype among joint isolates, indicating a higher frequency in joint-associated infections. One possible explanation for the changing prevalence of GT1.1 is differences in fitness under local epidemiological or management conditions, including antimicrobial exposure. A previous study reported that Italian strains belonging to *S. suis* serotype 9, sequence 16, showed lower ampicillin resistance compared to other *S. suis* sequences [44], and these same strains would be classified as GT2. In this study, *S. suis* serotypes 9, 2, 7, and 1 were the predominant serotypes recovered from clinical piglets; similar findings have been reported in previous studies [11,39,40,41,42,43].

It should also be noted that the overall prevalence of GT2 in Europe may be slightly overestimated, as the number of strains from different European countries and time was unequal. Because isolates were likely derived from convenience sampling, i.e., submissions from clinical cases for veterinary diagnostic laboratory testing, rather than systematic random surveillance, different periods or countries with outbreak activity or intense surveillance may be overrepresented relative to the baseline circulation, further limiting the generalizability of the genotype trends observed for the true European population structure. As an example, the highest number of isolates collected early on (2018 to 2021) and coming from the Netherlands had a relatively high GT2 prevalence (Figure 6). A total of 363 unknown serotype strains (Figure 7) were also investigated for the presence of the *recN* gene by PCR, whereas 13 were *recN* negative. The *recN* gene has been used to identify bacteria from the genera *Geobacillus* and *Streptococcus* [43,44], but it cannot determine *Streptococcus* species levels [45]. Potentially, the 13 strains may represent non-specific amplification, non-*Streptococcus suis* organisms, or organisms not belonging to the genus *Streptococcus*.

A qPCR assay was successfully developed and applied to identify different genotypes, GT1 and GT2, based on the Mac-1 domain classification and to further differentiate GT1.1 from GT1.2 strains based on the variable region outside the Mac-1 domain, since the Mac-1 of GT1 was very conserved and required an additional region for appropriate differentiation by qPCR. The assay was designed and implemented as a qualitative genotyping tool, classifying isolates as positive or negative for each genotype according to a predefined amplification curve and Ct criteria, rather than as a quantitative test of a target copy number. Under the assay conditions used in this study, positive control strains and positive test isolates consistently showed early amplification with low Ct values (typically around 10–20) and a single, reproducible melting peak at the expected temperature, with a variation of approximately ±2 °C, whereas negative controls remained without specific amplification or characteristic melting peaks.

The development of this assay enables implementation across veterinary diagnostic laboratories, facilitating large-scale genotyping and improving accessibility across regions. The assay was designed using a global sequence collection from GenBank, which provided a very good representation of Europe. To ensure run validity, each assay included both positive and negative controls. Nuclease-free water served as the negative control. Positive control strains for the three primer pairs were initially selected after testing several samples because they yielded the most reproducible results during qPCR testing in the authors’ diagnostic laboratories as well as in a previous study [25] and were subsequently confirmed as *Streptococcus* spp. by MALDI-TOF [46]. For strain documentation, the GT1 control strain (16085/3b) and the GT2 control strain (GD-0034) were selected from strains that had either been described in previous publications or were available in public sequence databases. The GT1.1 control strain 16085/3b has been described in detail in the literature, whereas the GT1.2 control strain (4-1-1-019) was used as a well-characterized field isolate provided by a reference diagnostic laboratory and is not, to our knowledge, linked to an individual peer-reviewed strain description. Positive control strains showed early amplification with low Ct values (approximately 18–21) and single, reproducible melting peaks at the expected temperatures, as summarized in Table 2, whereas non-template controls showed no specific amplification or melting peaks. Testing samples with this qPCR yielded detection trends across European countries and may be applicable in other countries and regions. One potential limitation of the global application of this qPCR assay is that some regions, e.g., Brazil, have a limited number of sequences (*n* = 1) and are underrepresented, which may lead to no detection or false-negative results, given the potential for genetic variability. ATCC positive/negative controls and an extended panel of non-*S. suis* strains were not tested in the present study, which represents a limitation of the current analytical validation. Therefore, potential cross-reactivity with closely related bacteria cannot be fully excluded at this stage, and future studies should assess cross-reactivity, specificity, sensitivity, and overall analytical performance using additional non-*S. suis* isolates and independent typing methods. To date, Ide*_Ssuis_* is the only experimentally characterized bacterial protease shown to specifically cleave intact porcine IgM of pigs [23,25]. Available publications do not establish that the Ide*_Ssuis_* gene is absolutely restricted to *S. suis*.

In line with the qualitative purpose of the assay, a formal limit-of-detection study was not performed, and analytical sensitivity across a dilution series of target DNA remains to be determined. Further testing and development of the qPCR assay for global applications is still needed, highlighting the importance of continued refinement and monitoring of *S. suis* genetic evolution. A total of 103 isolates could not be genotyped by qPCR. Of those 103 isolates, 39 had a serotype identified, indicating that another 64 isolates did not have a serotype detected and were not submitted to additional testing to rule in or rule out the presence of an *S. suis* isolate. The lack of further testing in these non-typable isolates reflects the retrospective nature of this study and the limited sample material in this dataset, which was primarily collected for Mac-1-based genotyping rather than comprehensive species identification. Future studies are planned to apply sequencing to non-typable isolates to further identify them to rule in or rule out *S. suis.* Future studies should therefore compare qPCR genotyping with a second independent method on both qPCR-negative and qPCR-positive isolates to confirm specificity, sensitivity, and overall analytical performance. Altogether, these findings provide a European overview of Mac-1 genotyping prevalence.

## 5. Conclusions

A genotype classification system based on the Mac-1 active domain and the most conserved region of Ide*_Ssuis_* was proposed. A Mac-1-based genotype classification differential qPCR assay was developed. A collection of European *S. suis* isolates were tested by qPCR for genotype classification. Based on the Mac-1 active domain of Ide*_Ssuis_*, two genotypes and two subgenotypes (GT1/GT2/GT1.1/GT1.2) were proposed. GT1.1 was the predominant subgenotype encountered in a collection of European *S. suis* isolates. Strains belonging to GT1.1 were the largest cluster and had a 97.1% mean amino acid sequence identity. Because the classification is paired with qPCR targets for genotype and subgenotype identification, it can be implemented broadly across institutions without requiring next-generation sequencing. The Mac-1-based genotype classification can complement serotyping and STs to classify *S. suis* strains and may help identify target strains for vaccine development.

## Figures and Tables

**Table 1:** Geographic and temporal contribution of European *S. suis* isolates to the dataset and sampling over time.

	2018	2019	2020	2021	2022	2023	Total
Austria		5			1		6
Belgium	77	135	159	106	81	79	637
France	207	273	259	262	287	285	1573
Germany	145	250	262	159	127	126	1069
Hungary		5	6	16	15	18	60
Netherlands	347	383	398	313	170	235	1846
Poland				9	8		17
Portugal	1	5	3	2			11
Romania						7	7
Slovakia				4			4
Spain	3	39	19	15	64	75	215
United Kingdom			13	6	8	7	34

**Table 2:** Primers used for GT1-, GT1.2-, and GT2-specific qPCR. Abbreviations: nt = nucleotide, aa = amino acid, bp = base pairs.

Primer	Sequence (5′-3′)	Region (Wild Type)	Fragment Size
GT1_Forward	actatacagcgccatttgagg	nt 500–520 (corresponding to aa 167 to 173)	130 bp
GT1_Reverse	tgttccaaccaccaatgtaccat	nt 607–629 (corresponding to aa 203 to 209	
GT2_Forward	ctgagcacgaagatagtagcc	nt 380–400 (corresponding to aa 128–133)	107 bp
GT2_Reverse	tctgtactgaccatcttcgct	nt 466–486 (corresponding to aa 156–162)	
GT1.2_Forward	cctatgtcagcagttggagaa	nt 1513–1533 (corresponding to aa 505–511)	173 bp
GT1.2_Reverse	actggtaagacgggttgattg	nt 1665–1685 (corresponding to aa 556–561)	

**Table 3:** Matrix for genotype assignment based on qPCR testing results.

Genotype Assignment	GT1 qPCR Result	GT1.2 qPCR Result	GT2 qPCR Result
GT1.1	Positive	negative	negative
GT1.2	Positive	Positive	negative
GT2	negative	negative	Positive

**Table 4:** Amino acid percentage homology within and between genotypes based on the Ide*_Ssuis_* Mac-1 domain.

	GT1	GT1.1	GT1.2	GT2
GT1	96.8			62.2
GT1.1		97.1	96.1	62.1
GT1.2		96.1	98.1	62.4
GT2	62.2	62.1	62.4	91.4

**Table 5:** Summary table for 427 unique Ide*_Ssuis_* sequences and each of their genome regions.

Groups	Minimum	Quantile 1	Median	Mean	Quantile 3	Maximum
Ide*_Ssuis_*	17	47.7	55.4	61.2	85.4	100
N-terminus & Signal peptide	16.3	65.9	70.3	71.3	96.7	100
Mac-1	58.1	93.2	96.2	88.7	97.6	100
Variable region	7.8	41.6	55.5	61.2	93.8	100
Repeated region	1.2	8.8	13.4	32.3	57	100
C-terminal variable region	2.4	28.8	31.6	52.9	92.9	100
Transmembrane region	18.2	59.1	63.6	68.8	95.5	100

**Table 6:** Repeatability of positive controls in the qPCR (*n* = 79); all negative controls remained negative.

	Cycle Threshold (Ct)			Melting Temperature		
	GT1.1	GT2	GT1.2	GT1.1	GT2	GT1.2
Mean	18.91	20.60	17.45	80.10	79.56	84.48
Standard deviation	1.23	1.38	2.12	0.41	0.38	0.30
Coefficient of variation (%)	6.5	6.7	12.1	0.5	0.5	0.4

**Table 7:** Comparison of Mac-1 domain-based genotype versus a previous [25] genotype classification system based on Ide*_Ssuis_*.

Item	This Study	Previous Study [25]
Targeted *S. suis* genome region	Mac-1 active domain of Ide*_Ssuis_*	Ide*_Ssuis_*
Initial set of *S. suis* candidate sequences	10,641	3111
Identified Ide*_Ssuis_*	4161	2586
Selection criteria	Broadened to remove: (a) Incomplete sequences with <800 aa; (b) sequences lacking a stop codon at the end of the coding sequence; (c) sequences containing ambiguous residues; (d) sequences lacking a predicted signal peptide, SignalP-6.0; (e) sequences lacking a predicted transmembrane (TM) segment; (f) sequences lacking an N-terminal methionine; (g) duplicated sequences.	Removed: (a) Duplicate sequences; (b) Incomplete sequences with <800 aa.
Retained Ide*_Ssuis_*	427	1999

## Data Availability

The original contributions presented in this study are included in the article/Supplementary Materials. Further inquiries can be directed to the corresponding author.

## Supplementary Materials

The following supporting information can be downloaded at: https://www.mdpi.com/article/10.3390/vetsci13080807/s1, Table S1: Reference list for 427 unique Mac-1 domain sequences.

## Abbreviations

The following abbreviations are used in this manuscript:

aa	Amino acid
ATCC	American Type Culture Collection
bp	Base pairs
CPS	Capsular polysaccharides
CI	Confidence interval
DNA	Deoxyribonucleic acid
GT1	Genotype 1
GT2	Genotype 2
GT1.1	Genotype 1 subgenotype 1
GT1.2	Genotype 1 subgenotype 2
IdeS	Endoprotease IdeS of *S. pyogenes*
IgA	Immunoglobulin A
IgG	Immunoglobulin G
IgM	Immunoglobulin M
MAFFT	Multiple alignment using fast Fourier transform
MALDI-TOF	Matrix-assisted laser desorption/ionization–time-of-flight mass spectrometry
MLST	Multilocus sequence typing
NCBI	National Center for Biotechnology Information
nt	Nucleotide
RAPT	Read Assembly and Annotation Pipeline Tool
ST	Sequence type
*S. suis*	*Streptococcus suis*
qPCR	Qualitative polymerase chain reaction
